# Stealth Magnetoliposomes Based on Calcium-Substituted Magnesium Ferrite Nanoparticles for Curcumin Transport and Release

**DOI:** 10.3390/ijms21103641

**Published:** 2020-05-21

**Authors:** Beatriz D. Cardoso, Ana Rita O. Rodrigues, Bernardo G. Almeida, Carlos O. Amorim, Vítor S. Amaral, Elisabete M. S. Castanheira, Paulo J. G. Coutinho

**Affiliations:** 1 Centre of Physics (CFUM), University of Minho, Campus de Gualtar, 4710-057 Braga, Portugal; beatrizdiascardoso94@gmail.com (B.D.C.); ritarodrigues@fisica.uminho.pt (A.R.O.R.); bernardo@fisica.uminho.pt (B.G.A.); ecoutinho@fisica.uminho.pt (E.M.S.C.); 2 Physics Department and CICECO, University of Aveiro, Campus de Santiago, 3810-193 Aveiro, Portugal; amorim5@ua.pt (C.O.A.); vamaral@ua.pt (V.S.A.)

**Keywords:** magnetic nanoparticles, calcium-substituted magnesium ferrites, magnetoliposomes, curcumin release, cancer therapy

## Abstract

Despite the promising pharmacological properties of curcumin, the transport and effective release of curcumin is still a challenge. The advances in functionalized nanocarriers for curcumin have also been motivated by the anticancer activity of this natural compound, aiming at targeted therapies. Here, stealth (aqueous and solid) magnetoliposomes containing calcium-substituted magnesium ferrite nanoparticles, Ca_x_Mg_1−x_Fe_2_O_4_ (with *x* = 0.25, 0.50, 0.75) were developed as nanocarriers for curcumin. The magnetic nanoparticles exhibit superparamagnetic properties and crystalline structure, with sizes below 10 nm. The magnetoliposomes based on these nanoparticles have hydrodynamic diameters around or below 150 nm and a low polydispersity. The influence of an alternating magnetic field (AMF) on drug release over time was evaluated and compared with curcumin release by diffusion. The results suggest the potential of drug-loaded magnetoliposomes as nanocarriers that can be magnetically guided to the tumor sites and act as agents for a synergistic effect combining magnetic hyperthermia and controlled drug release.

## 1. Introduction

Curcumin is a polyphenolic compound that can be extracted from Turmeric (*Curcuma longa*). This phytochemical is a liposoluble compound that has been showing a wide range of promising pharmacological properties, which include anti-inflammatory, antioxidant, antidiabetic, antimicrobial, and anticancer actions [1,2,3,4]. The consumption of this compound at levels up to 20 mg as a food additive is recognized as safe by the US Food and Drug Administration (FDA) [5]. However, curcumin is chemically unstable, which is mainly related to solvolysis and oxidative degradation resulting in the production of some degradation products (e.g., vanillin, ferulic acid, and feruloylmethane). It is also sensitive to type I and type II reactions with molecular oxygen resulting in photochemical degradation [6]. In addition, it presents a low water solubility (456 μg/L), poor absorption, and rapid metabolism and elimination, which limit its biomedical use and therapeutic effect, justifying the lack of success in clinical trials [7,8]. In the last years, the scientific community has been focused on the development of strategies that aim to improve curcumin’s ADMET properties (absorption, distribution, metabolism, excretion, and toxicology) and its specificity. Some of these drawbacks can be minimized by curcumin encapsulation in suitable nanosystems.

The use of drug delivery systems is one of the most employed and effective strategies to address drug bioavailability issues by protecting and carrying payloads to the target site. The nanocarriers include, for instance, micelles, microemulsions, nanogels, polymeric nanoparticles, carbon nanotubes, magnetic nanoparticles, and liposomes. Liposomes are lipid layered vesicles which can encapsulate a wide range of compounds and improve their solubility. They show a high biocompatibility, low toxicity, and low immunogenicity. The liposomes preparation method and variations in composition allow to modify their inherent features, such as size, circulation time in biological conditions, responsiveness to specific external and internal stimuli, and to control payload release. All the modifications must be accurately developed in order to take advantage of the uniqueness of cancer physiological features, since they include chemical (such as enzyme concentration and pH) and structural (capillary structure) differences compared to normal cells/tissues. Considering the leaky nature of tumor blood vessels, as a result of the presence of gaps within the range of 100 to 780 nm between the endothelial cells of tumor capillaries and the lack of lymphatic drainage, liposomes are an effective strategy for passive targeting. This phenomenon, known as the enhanced permeability and retention (EPR) effect, allows the extravasation of liposomes smaller than 400 nm, but it is more effective at sizes below 200 nm [9,10]. However, it is necessary to ensure that the liposomes are not rapidly captured by the liver and spleen phagocytic cells and pass as many times as possible through the target site. Polyethylene glycol (PEG) is a polymer that forms a hydrophilic shield on the liposome surface, which prevents liposome uptake by the reticuloendothelial system (RES), contributing for a prolonged systemic circulation. As a result, nonmodified liposomes and PEGylated liposomes have gained attention as an improved therapeutic drug delivery system for cancer therapy, resulting in some FDA-approved liposomal formulations. However, the associated defective blood perfusion in tumors leads to a low pH and oxygen (hypoxia) inside the solid tumors and surrounding tissues. These conditions difficult the therapeutic effect, either of radiotherapy (based on the creation of reactive oxygen species) or chemotherapy (since the drugs are not able to concentrate in the tumor at the aimed therapeutic dosage). Therefore, many efforts are being made to further improve liposomes’ therapeutic effectiveness.

Superparamagnetic nanoparticles are single domain magnetic materials with heating ability under an alternating magnetic field (AMF), and can be entrapped in liposomes (forming magnetoliposomes) and used as mediators for magnetic hyperthermia. This approach brings complementary advantages to lipid vesicles, since it allows one to selectively direct the nanosystems to the tumor by a magnetic gradient, a further increase of chemotherapeutic drugs concentration on tumor, and to directly overheat cancer cells under an AMF.

Even though magnetite and maghemite are the most studied nanoparticles for magnetic hyperthermia, they have been associated with a tendency to react with oxygen which results in the production of free radicals as reactive oxygen species (ROS), leading to oxidative damage in the human body [11]. In order to be suitable for entrapping in liposomes and use in magnetic hyperthermia, magnetic nanoparticles must be biocompatible. The synthesis of particles without transition metals has been exploited. Spinel ferrites, MFe_2_O_4_ (where M = Fe, Mn, Zn, Co, Mg, Cu, Ni) present unique electric, optical, and magnetic properties. As a result, they have been applied, for instance, for contrast enhancement of magnetic resonance imaging and as magnetic carriers for drug delivery [12]. Magnesium ferrite (MgFe_2_O_4_) is a class of compounds that crystalize into an inverse spinel structure with tetrahedral (A) and octahedral (B) sites, where cations distribution occurs. The magnitude of magnetization is highly sensitive to the synthesis methodology, to the distribution of magnetic ions in tetrahedral and octahedral sites, and to grain size and shape. The substitution of transition and diamagnetic metal ions is a strategy that can be followed to improve the magnetization of spinel ferrites [13]. Calcium ferrite (CaFe_2_O_4_) nanoparticles are biocompatible, eco-friendly, and present a high thermal stability [14]. Calcium-substituted magnesium ferrite nanoparticles (Ca_x_Mg_1−x_Fe_2_O_4_) not only can promote an increase in ferrite biocompatibility, but also improve the magnetic properties [15,16]. It is reported that upon doping with nonmagnetic Zn^2+^ ions, magnesium ferrite nanoparticles showed an enhanced magnetization as a result of changes in ion site distribution [17,18]. 

In this work, three different proportions of calcium and magnesium were used to synthesize Ca_x_Mg_1−x_Fe_2_O_4_ nanoparticles (*x* = 0.75, 0.25, 0.50), and their optical, structural, morphological, and magnetic properties were characterized. The mixed ferrites were included in magnetoliposomes, forming the magnetic core, either within an aqueous phase (aqueous magnetoliposomes, AMLs) or surrounded by a lipid bilayer without water (solid magnetoliposomes, SMLs). Curcumin was encapsulated in these novel nanosystems and its release rate was evaluated under an alternating magnetic field. The influence of magnetoliposomes PEGylation in curcumin location and release was also investigated. The developed nanosystems are promising for applications as curcumin nanocarriers for synergistic cancer therapy (using a combined approach of magnetic hyperthermia and chemotherapy), considering the already reported antitumor properties of curcumin.

## 2. Results and Discussion

### 2.1. Fluorescence Properties of Curcumin

Curcumin is a phytochemical that is soluble in both polar and nonpolar organic solvents and insoluble in water at neutral and acidic pH. Its absorption spectrum shows two strong bands, one in the visible region (~410–430 nm) and another in the UV region (maximum at 265 nm). The electronic energy levels occur between a bonding or lone-pair orbital and an unfilled nonbonding or antibonding orbital [19]. In aqueous solution, curcumin absorption decreases, not only as a result of a direct reduction in the number of absorption centers, but also due to curcumin degradation in water by a reaction at the keto-enol group [20]. The diketo group can exist in different conformers, since it exhibits keto-enol tautomerism [21]. Unlike curcumin absorption maximum, the fluorescence emission intensity and the position of emission band is highly sensitive to the nature of solvent [22]. Curcumin does not present fluorescence emission in aqueous media. This quenching effect by water has been ascribed to a reaction between water (as an electron donor) and curcumin, which results in a more stable and nonfluorescent complex with lower S_0_ energy [23].

In this investigation, the fluorescence properties of curcumin were used to monitor curcumin encapsulation and release from the developed magnetoliposomes. 

### 2.2. Nanoparticles Characterization

#### 2.2.1. UV–Visible Absorption Spectra

**Figure 1:**
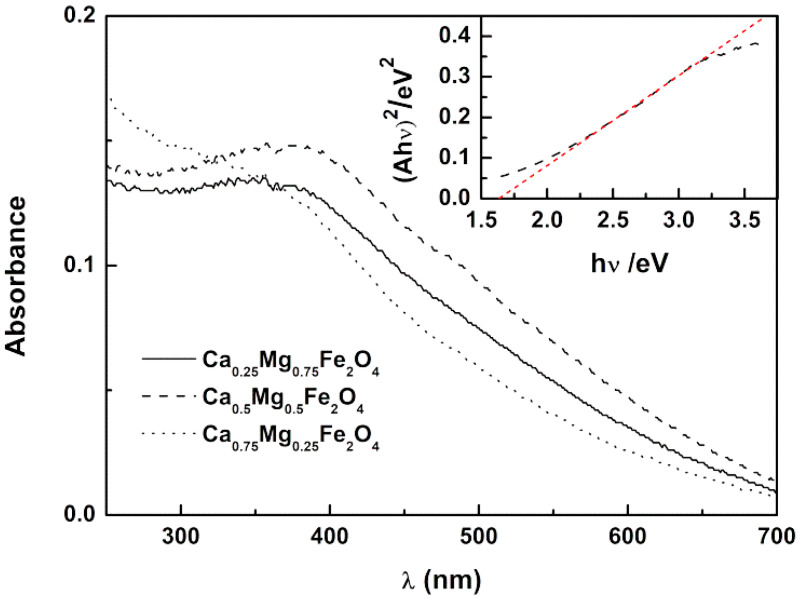
Absorption spectra of Ca_0.25_Mg_0.75_Fe_2_O_4,_ Ca_0.50_Mg_0.50_Fe_2_O_4_, and Ca_0.75_Mg_0.25_Fe_2_O_4_ magnetic nanoparticles. Inset: Tauc plot for Ca_0.50_Mg_0.50_Fe_2_O_4_ nanoparticles, as an example (red line is the linear fitting).

The optical absorption and band gap energy of the prepared nanoparticles were investigated by UV–Vis spectroscopy, which is an indicator of their purity and composition. The prepared nanoparticles showed a broad absorption in the UV–visible wavelength range, between 250–650 nm (Figure 1). This wide absorption range is a key feature for the objectives of this work, as it reveals that nanoparticles may quench fluorescence emission of curcumin when encapsulated in magnetoliposomes. This effect has already been reported for drug-loaded magnetoliposomes based on magnesium ferrite [22] and calcium ferrite nanoparticles [24].

The UV–Visible absorption spectra of the synthesized Ca_x_Mg_1−x_Fe_2_O_4_ nanoparticles (Figure 1) allow obtaining the optical band gap values using a Tauc Plot, by the equation:

(1) $${\left(\alpha h\nu \right)}^{n}\propto \left(h\nu -E_{g}\right)$$

where α is the absorption coefficient, *n* is an exponent that depends on the nature of the transition (being *n* = 2 for a direct semiconductor and *n* = 1/2 for an indirect one), and *E_g_* is the optical band gap.

The Ca_0.25_Mg_0.75_Fe_2_O_4_, Ca_0.50_Mg_0.50_Fe_2_O_4_, and Ca_0.75_Mg_0.25_Fe_2_O_4_ nanoparticles are direct semiconductors, as a linear relation was only obtained for *n* = 2. From the Tauc plots, the band gaps of the different nanoparticles were estimated (Tauc plot for Ca_0.50_Mg_0.50_Fe_2_O_4_ is shown as example in inset of Figure 1). Band gaps of 1.51 eV, 1.65 eV, and 1.91 eV were obtained for Ca_0.25_Mg_0.75_Fe_2_O_4_, Ca_0.50_Mg_0.50_Fe_2_O_4_ and Ca_0.75_Mg_0.25_Fe_2_O_4_ nanoparticles, respectively. The band gap values show a tendency to increase as the calcium ratio increases in these mixed ferrite nanoparticles. These results agree with the previously reported values of 1.41 eV for MgFe_2_O_4_ [22] and 2.19 eV for CaFe_2_O_4_ [24], similar to the value of 1.90 eV reported by Kim et al. for calcium ferrite [25]. 

#### 2.2.2. X-Ray Diffraction (XRD) Measurements

**Figure 2:**
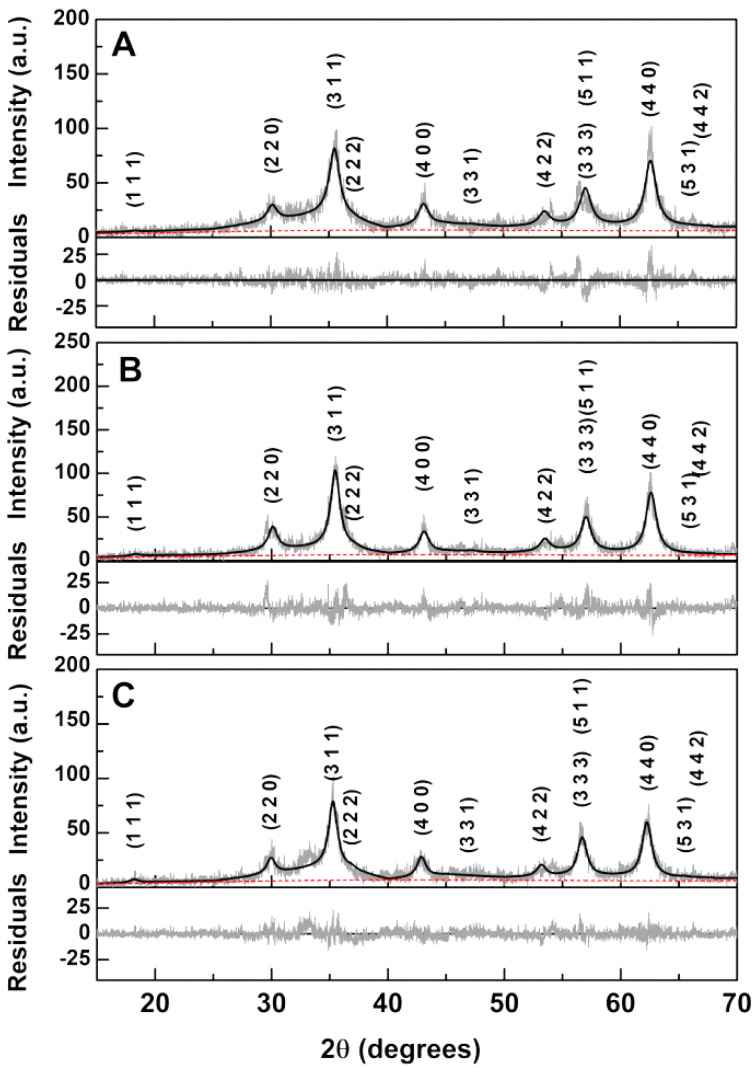
XRD patterns with Miller indices of the synthesized calcium-substituted magnesium ferrite nanoparticles. (**A**): Ca_0.25_Mg_0.75_Fe_2_O_4_; (**B**): Ca_0.50_Mg_0.50_Fe_2_O_4_; (**C**): Ca_0.75_Mg_0.25_Fe_2_O_4_. Gray lines: experimental patterns; black lines: fitted patterns; red dashed lines: background.

XRD measurements confirmed the synthesis of the calcium-substituted magnesium ferrite nanoparticles as, after a calcination process at 600 °C, a pure crystalline ferrite phase corresponds to the observed diffraction peaks (space group Fd$\bar{3}$m, CIF 9000926), marked by their indices (Figure 2). 

For CaFe_2_O_4_ nanoparticles, we have previously used an inversion degree of *i* = 0.85 [24], and for MgFe_2_O_4_, a value of *i* = 0.83 was obtained [22]. Thus, for the ferrites under study, a value of *i* = 0.85 was chosen and full Rietveld analysis using *FullProf* software suite (version 5.8, J. Rodríguez-Carvajal, Lab. Léon Brillouin, Gif sur Yvette, France) [26] was then performed, with the main results reported in Table 1 and the resulting fits presented in Figure 2. In a previous study [27], we found it important to use microabsorption correction [28]. The fits obtained with this correction exhibit the same quality as the ones obtained by optimizing the overall isothermal Debye–Waller factor, B_over_, which resulted in a negative value, having no physical meaning. Crystallite sizes of 5.8 nm, 6.4 nm, and 7.0 nm were estimated by the Scherrer equation for the Ca_0.25_Mg_0.75_Fe_2_O_4_, Ca_0.50_Mg_0.50_Fe_2_O_4_, and Ca_0.75_Mg_0.25_Fe_2_O_4_ nanoparticles, respectively. The lattice parameter is seen to increase with Ca content and more significantly when the Ca percentage is 75% (Table 1). 

#### 2.2.3. Sedimentation Kinetics

**Figure 3:**
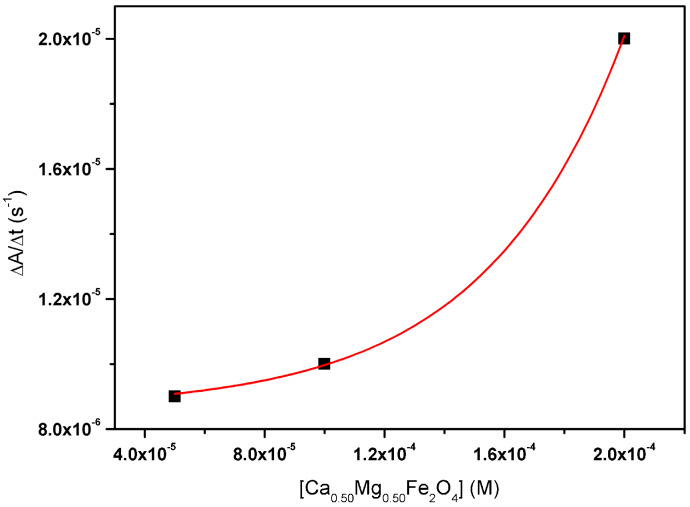
Sedimentation kinetics of Ca_0.50_Mg_0.50_Fe_2_O_4_ nanoparticles.

The sedimentation kinetics of the synthesized calcium-substituted magnesium ferrite nanoparticles was followed by measuring the UV–Visible absorption of nanoparticle dispersions as a function of time. The results for Ca_0.50_Mg_0.50_Fe_2_O_4_ NPs, obtained as the slopes of the absorption variation function over time, for three different NP concentrations, are shown as an example (Figure 3). A similar behavior was observed for all nanoparticles, with high stability for concentrations below 1 × 10^−4^ M and sedimentation exponential growth for higher concentrations. However, after one hour, the nanoparticles remained extremely dispersed, in percentages of 85%, 82%, and 97% for Ca_0.25_Mg_0.75_Fe_2_O_4_, Ca_0.50_Mg_0.50_Fe_2_O_4_, and Ca_0.75_Mg_0.25_Fe_2_O_4_, respectively. This is a positive indication that the stabilities of the nanoparticles’ aqueous dispersions are suitable for biomedical applications.

#### 2.2.4. Transmission Electron Microscopy (TEM)

**Figure 4:**
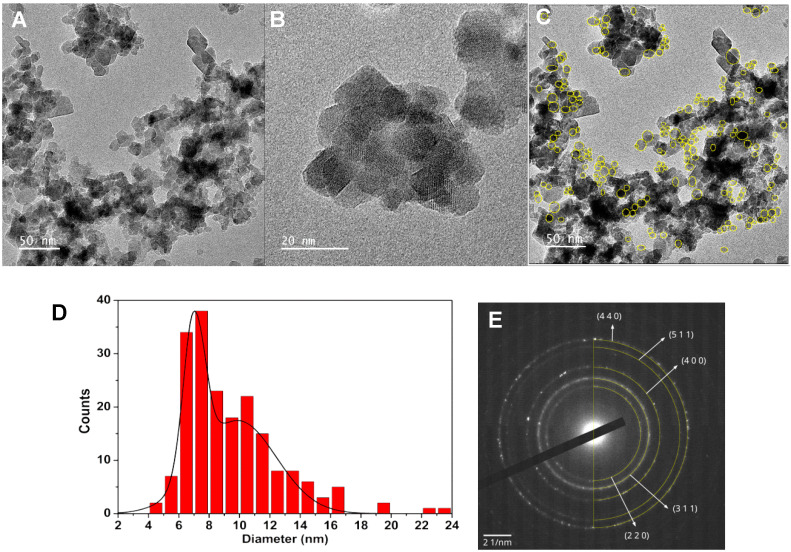
(**A**,**B**): Transmission electron microscopy images of Ca_0.50_Mg_0.50_Fe_2_O_4_ nanoparticles with different magnifications. (**C**): Image (**A**) with outlined nanoparticles. (**D**): Size histogram of image (**A**). (**E**): Small area electron diffraction (SAED) image with indexed diffraction planes.

Transmission electron microscopy (TEM) images, together with small area electron diffraction (SAED), for Ca_0.50_Mg_0.50_Fe_2_O_4_ nanoparticles are shown in Figure 4. Upon manual outline of 193 particles (Figure 4C), the size was determined considering an equivalent area circle. The analysis of the corresponding size histogram (Figure 4D) resulted in two populations, with sizes 7.0 ± 0.7 nm and 9.9 ± 2.5 nm. The larger population probably corresponds to image projections of nanoparticle aggregates. The deviation from sphericity was analyzed by comparing the particle’s perimeter with that calculated with the equivalent area circle. The majority of the particles have 0.95 sphericity, while a 5% fraction shows a value of 0.84. SAED results (Figure 4E) are compatible with a spinel ferrite crystal structure, as seen from the added semicircles. These correspond to spinel structure d-spacings calculated using the same lattice constant that was obtained from XRD Rietveld analysis (8.3915 Å). 

#### 2.2.5. Magnetic Properties 

The magnetic properties of calcium-substituted magnesium ferrite nanoparticles (Ca_x_Mg_1−x_Fe_2_O_4_) emphasize that the partial substitution of magnesium by calcium in the ferrite structure has nonlinear effects on the maximum magnetization. The overall magnetization is sensitive to the distribution of Fe^3+^ ions in A (tetrahedral) and B (octahedral) sites, as the magnetic moment of Fe^3+^ is 5 µ_B_, while Ca^2+^ and Mg^2+^ are nonmagnetic. Furthermore, the magnetization directly depends on the Ca^2+^ concentration, a large cation whose radius is near the threshold of ~1 Å, with high implications on the distribution of magnetic ions in interstitial sites. At low concentration values, Ca^2+^ ions show a preferred occupancy at A-sites, with (1 – *x* − *y*) Fe^3+^ ions on the A-site and (1 + *x* + *y*) Fe^3+^ ions on the B-site, where *y* and *x* represent the probability of migration of a small fraction of Mg^2+^ and Ca^2+^ ions to A-sites, respectively [29]. Thus, the distribution of calcium-substituted magnesium ferrite, for low Ca^2+^ content, takes the form $\left({\mathrm{Ca}}_{x}^{2+}{\mathrm{Mg}}_{y}^{2+}{\mathrm{Fe}}_{1-x-y}^{3+}\right)\left[{\mathrm{Ca}}_{x}^{2+}{\mathrm{Mg}}_{y}^{2+}{\mathrm{Fe}}_{1+x+y}^{3+}\right]O_{4}^{2-}$, where the curve and square brackets indicate tetrahedral (A-sites) and octahedral (B-sites) sublattice, respectively. Hence, the net magnetization |𝑀| = |𝑀_B_| − |𝑀_A_|, according to Neel’s theorem of sublattice [30], increases with the inclusion of Ca^2+^, due to the increasing number of Fe^3+^ ions in B-sites and decreasing amount in A-sites. For larger calcium concentration, *x* > 0.05, it is reported that Ca^2+^ ions migrate to B-sites and the distribution then take the form $\left({\mathrm{Ca}}_{x-y}^{2+}{\mathrm{Fe}}_{1+x-y}^{3+}\right)\left[{\mathrm{Mg}}_{1-x}^{2+}{\mathrm{Ca}}_{y}^{2+}{\mathrm{Fe}}_{1+x-y}^{3+}\right]O_{4}^{2-}$ [31]. Therefore, an increase of Ca^2+^ ions will lead to a decrease of Fe^3+^ ions in B-sites and an increase in A-sites, weakening the overall magnetization of the whole lattice [29,30].

**Figure 5:**
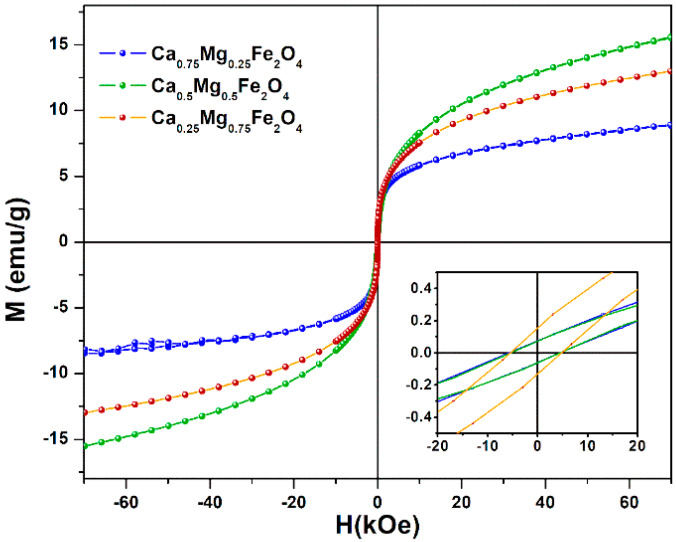
Magnetization hysteresis loops of calcium-substituted magnesium ferrite nanoparticles measured at room temperature. Inset: Enlargement of the loops in the low field region.

Figure 5 shows the magnetization hysteresis cycles of synthesized calcium-substituted magnesium ferrite nanoparticles, displaying the relationship between the induced magnetic moment and the applied magnetic field (H) at room temperature. The magnetic properties, saturation magnetization (M_s_), remnant magnetization (M_r_), and coercive field (H_c_) are presented in Table 2. Higher saturation magnetization values were obtained for Ca_0.25_Mg_0.75_Fe_2_O_4_ and Ca_0.5_Mg_0.5_Fe_2_O_4_ nanoparticles, with 12.98 Oe and 15.63 Oe, respectively. A lower value of M_s_, 8.83 Oe, was observed for Ca_0.75_Mg_0.25_Fe_2_O_4_, indicating that Ca^2+^ ions migrated to B-sites, leading to a decrease of Fe^3+^ ions in B-sites (and corresponding increase on A-sites), resulting in a lower magnetization. 

In any case, these values compare well with the previously reported maximum magnetization for MgFe_2_O_4_ (16.16 emu/g [22]) and for CaFe_2_O_4_ (12.81 emu/g [24]) nanoparticles obtained by the same method of preparation. 

The superparamagnetic behavior of the nanoparticles was investigated, taking into account the magnetic squareness value (ratio between the remnant and saturation magnetizations). If this value is below 0.1, more than 90% of the magnetization is lost upon the removal of the applied magnetic field. Here, the calculated magnetic squareness values indicate that the synthesized ferrite nanoparticles are superparamagnetic at room temperature, having low coercivity and remnant magnetizations (Table 2). 

The substitution of 50% magnesium by calcium in the ferrite structure allows improved magnetic properties relative to calcium ferrite and an enhanced biocompatibility relative to magnesium ferrite, as calcium ferrite is reported as highly biocompatible in studies of cell viability [31]. All the prepared mixed nanoparticles exhibit superparamagnetic properties and avoid undesirable oxidation and production of reactive oxygen species (ROS) in mammalian cells by iron oxide nanoparticles, which cause protein and DNA damage and inflammatory responses [11,32,33,34].

### 2.3. Characterization of Magnetoliposomes

To the best of our knowledge, aqueous and solid magnetoliposomes based on the developed calcium/magnesium mixed ferrite nanoparticles were prepared for the first time. These new nanosystems were tested as magnetic nanocarriers for curcumin. 

#### 2.3.1. Aqueous Magnetoliposomes

Aqueous magnetoliposomes were obtained by entrapping magnetic nanoparticles in liposomes of the natural lipid mixture egg–phosphatidylcholine. AMLs can be used as nanocarriers for both hydrophilic and hydrophobic drugs, but their magnetic properties are poorer than the ones of solid magnetoliposomes (that do not present an aqueous inner volume) due to the diamagnetic contribution of water in AMLs [35,36].

The hydrodynamic size, polydispersity index (PDI), and zeta potential values were measured by dynamic and electrophoretic light scattering (Table 3), indicating generally monodisperse systems (low PDI values) with sizes below 150 nm, suitable for passive targeting through the EPR effect. These sizes are similar to the ones obtained for magnetoliposomes containing calcium ferrite nanoparticles [24], and remain constant after two weeks of storage. Zeta potential measurements point to a negative surface charge of the nanostructures (Table 3).

**Figure 6:**
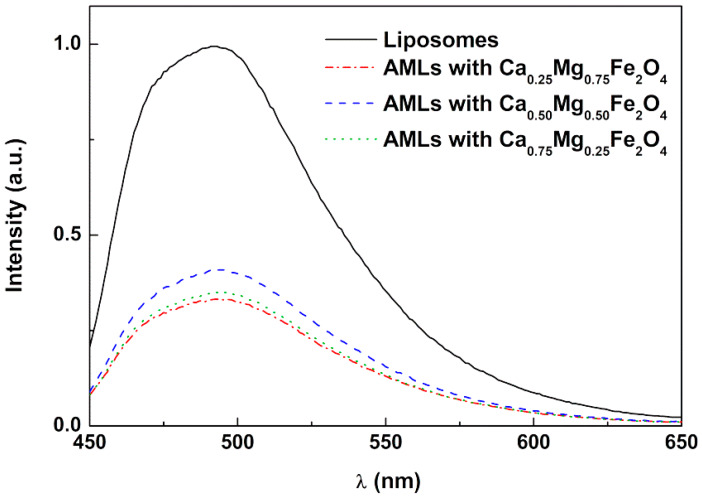
Fluorescence spectra of curcumin encapsulated in liposomes and aqueous magnetoliposomes based on calcium-substituted magnesium ferrite nanoparticles.

The curcumin encapsulation in AMLs based on the three different ferrite nanoparticles was investigated by fluorescence emission (Figure 6). The fluorescence spectra were compared between liposomes (of the same lipid composition but without magnetic nanoparticles) and AMLs, both encapsulating curcumin. The emission spectra allow confirming the incorporation of curcumin in vesicles, exhibiting a quenching effect of drug fluorescence in the presence of magnetic nanoparticles (as expected from the wide wavelength range of nanoparticles absorption). No significant shifts in the band of curcumin were noted between liposomes and AMLs, indicating a similar environment felt by curcumin in the different nanosystems. 

In order to prevent opsonization and allow shielding from proteolytic enzymes, increasing the retention time in the circulation system, PEGylation of Egg–PC aqueous magnetoliposomes was carried out with 5% DSPE-PEG-2000. While it has been reported that coating with PEG is effective in increasing the circulation time of oleic acid-coated magnetite nanoparticles [37] and also of magnetoliposomes [38], it was also recently argued that PEGylation can reduce the interaction of targeted magnetoliposomes with cells, with a decrease in the degree of internalization [39]. Nevertheless, PEGylation remains the main strategy to improve the circulation time of lipid nanocarriers (known as stealth liposomes). 

Fluorescence anisotropy measurements of curcumin (Table 4) were determined. The values in Egg–PC liposomes and glycerol are presented for comparison, as in this highly viscous medium (η = 993.4 cP at room temperature [40]), curcumin molecules rotate very slowly and the anisotropy value approaches the fundamental anisotropy of the fluorophore [41]. From the anisotropy values, one can observe that the values in aqueous magnetoliposomes are similar to those in liposomes, proving curcumin’s location in the lipid membrane, as expected from its hydrophobic character. The Egg–phosphatidylcholine (PC) bilayer is expected to be more fluid than glycerol, considering its lipid composition (main components are 16:0 PC, 18:0 PC and 18:1 PC), justifying lower anisotropy values for curcumin (higher degree of rotation). Lipid membranes have microviscosities that depend on their lipid composition, but generally lying in the range of 100–200 cP [42,43]. The addition of a superficial PEG layer seems to not influence the degree of rotation of curcumin in AMLs, as inferred from the similar anisotropy value.

**Figure 7:**
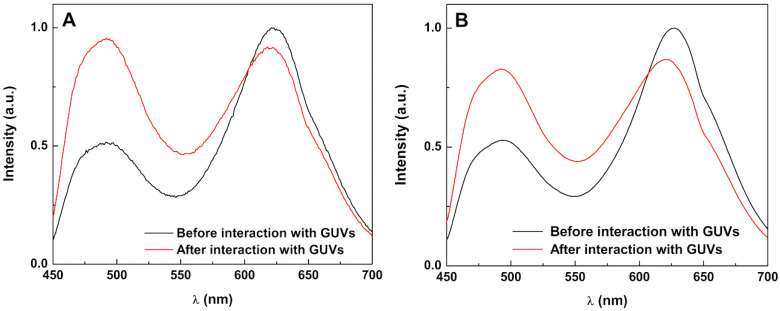
Fluorescence spectra (λ_exc_ = 440 nm) of aqueous magnetoliposomes (AMLs) containing Ca_0.25_Mg_0.75_Fe_2_O_4_ nanoparticles (as an example), labelled with both curcumin (2 × 10^−6^ M) and Nile Red (2 × 10^−6^ M), before and after interaction with giant unilamellar vesicles (GUVs). (**A**) Aqueous magnetoliposomes (AMLs) of Egg–PC; (**B**) AMLs of Egg–PC/DSPE-PEG2000 (0.95:0.05).

The hydrophobic nature of curcumin causes difficulties in its release from (magneto)liposomes. Therefore, it is important to assess whether the developed nanosystems can fuse with model membranes, allowing drug release. This capability was investigated by FRET, using curcumin as energy donor and the lipid probe Nile Red as an energy acceptor [22], both included in the magnetoliposomes. Giant unilamellar vesicles (GUVs) [44,45] were used as models of cell membranes. Exciting only the donor curcumin, a strong fluorescence band due to Nile Red emission is observed (maximum emission wavelength at ca. 625 nm). This is a consequence of the nonradiative energy transfer (FRET) from curcumin to Nile Red (Figure 7). After interaction with GUVs, the donor (curcumin) fluorescence increases and the acceptor emission band decreases, proving the decrease of efficiency of FRET process and, consequently, confirming the membrane fusion between aqueous magnetoliposomes and the model membranes. PEGylated magnetoliposomes (Figure 7B) also present fusion capabilities, but to a slightly lower extent, as inferred from the lower increase of donor fluorescence (maximum emission around 500 nm) after interaction with GUVs.

#### 2.3.2. Solid Magnetoliposomes

Regarding solid magnetoliposomes (SMLs) and taking into account the hydrophobic character of curcumin, a temperature-sensitive lipid, DPPC, with melting transition temperature of 41 °C [46], was considered as a good choice, as this transition temperature is near the ones used in mild hyperthermia therapy [47]. Upon melting, the lipid bilayer chains experience an increase in fluidity (attaining liquid-crystalline phase), with the possibility of enhanced drug release into cell membranes. 

To confirm the formation of the lipid bilayer around magnetic nanoparticles clusters, a FRET assay was employed (Figure S1 in Supplementary Material), where the labeled lipid NBD-C_12_-HPC was inserted in the inner lipid layer of SMLs and the lipid Rhodamine B-DOPE (rhodamine B as the acceptor) was incorporated in the outer lipid layer, NBD being the donor and Rhodamine B the acceptor, as described in previous works [22,24,36]. FRET efficiencies were calculated (Table 5), allowing determination of donor-acceptor distances (through Equations S1–S4 in Supplementary Material, Table 5). The different Förster radius values obtained reflect differences in the fluorescence quantum yield of the donor, as a result from a distinct quenching effect by the several nanoparticles. The donor–acceptor distances confirm the formation of the double lipid layer in solid magnetoliposomes (cell membranes have a thickness of 7 nm to 9 nm [48]). 

Solid magnetoliposomes size, polydispersity, and zeta potential were measured by DLS/ELS, revealing hydrodynamic diameters around or below 150 nm and low PDI (Table 6). The size of this type of nanocarriers has a tendency to increase with the calcium content of the nanoparticles. As for AMLs, the size values remained constant for two weeks and are adequate for passive targeting through the EPR effect. Zeta potential values (Table 6) are slightly more negative than the ones obtained for AMLs. 

To confirm curcumin’s location in SMLs, fluorescence anisotropy measurements were also performed (Table 7). The measurements were carried out at room temperature, where DPPC is in the rigid gel phase, and at 55 °C, above the melting transition temperature of DPPC (liquid-crystalline phase). It can be observed that above the transition temperature, the anisotropy of curcumin is significantly lower as a result of the increase in fluidity of curcumin microenvironment, pointing to a location in the lipid membrane of SMLs with or without a PEG layer. The lower values of anisotropy in SMLs at room temperature, relative to AMLs (Table 4), may reflect a deep penetration of curcumin in lipid membrane of SMLs, considering that the membrane fluidity decreases from the liposome surface to the interior [49,50]. In PEGylated SMLs, the higher anisotropy at 25 °C may indicate a more exterior location of curcumin. 

**Figure 8:**
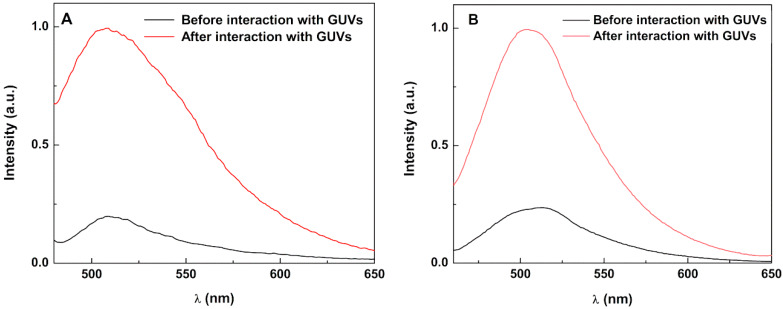
Fluorescence spectra (λ_exc_ = 440 nm) of curcumin in solid magnetoliposomes (SMLs) containing Ca_0.25_Mg_0.75_Fe_2_O_4_ nanoparticles (as an example) before and after interaction with GUVs. (**A**) SMLs of DPPC; (**B**) SMLs of DPPC/DSPE-PEG.

To investigate the interaction of SMLs with model membranes (GUVs), FRET assays were not used. Usually, FRET is not observed due to the strong quenching of both donor and acceptor emissions by the cluster of magnetic nanoparticles [22,24], which is very close to the fluorophores (due to the absence of the aqueous pool). Instead, if interaction with GUVs occurs, an unquenching effect should be observed. Taking advantage of curcumin’s intrinsic fluorescence, the assay was performed using curcumin-loaded magnetoliposomes. Upon interaction with GUVs, a significant rise in curcumin fluorescence was observed (unquenching effect), proving the increase in distance between the nanoparticles and the fluorescent drug due to membrane fusion (Figure 8). The effect is also observed for stealth (PEGylated) SMLs (Figure 8B). Although curcumin can bind through its enolic group to divalent metal ions on nanoparticle surfaces (originating an emission quenching) [51], the unquenching effect after interaction with GUVs demonstrates that curcumin is released from the nanocarriers.

#### 2.3.3. Curcumin Release Profile

The release behavior of curcumin from loaded SMLs towards model membranes (GUVs) was studied in the presence and absence of an alternating magnetic field (AMF) to evaluate the drug release profile from magnetoliposomes under magnetic stimulus. The influence of PEGylation was also investigated. The effect of AMF on curcumin release was investigated by exposing curcumin-loaded SMLs based on Ca_0.50_Mg_0.50_Fe_2_O_4_ nanoparticles to a magnetic field with intensity of 2.98 mT and frequency of 10,000 kHz. To be considered for therapy purposes, restriction limits have been set for tolerance and safety of patients. Originally, the Atkinson–Brezovich limit was asset as field-frequency product (H·f) of 4.85 × 10^8^ A·m^−1^·s^−1^. However, in practice, a higher limit is used (H·f ≤ 5 × 10^9^ A·m^−1^·s^−1^), as it depends on the area of application in the body [52]. Hence, the field and frequency values used are in accordance with the restriction limits, H·f = 2.38 × 10^9^ A·m^−1^·s^−1^ (H = 2.9 mT = 2379.087 A·m^−1^ and f = 1000 kHz = 10^6^ s^−1^); therefore, the field and frequency product is below 5 × 10^9^ A·m^−1^·s^−1^.

Three independent experiments were carried out to determine the average release ratio and corresponding standard deviation. The obtained experimental data were analyzed by using the nonlinear Korsmeyer–Peppas model (Table 8) [53,54,55,56]. Drug transport constants (*K*) and transport exponents (*n*) were determined by fitting to the Korsmeyer–Peppas equation (Equation (2)):

(2) CtC0=K·tn

where *C*_0_ and *C*_t_ are the concentrations at time 0 and *t*, respectively, *K* is the rate constant and *n* is the transport exponent. When *n* < 0.45, the release mechanism is diffusion-controlled (Fickian release), 0.45 < *n* < 0.89 indicates a combination of diffusion and erosion drug release (non-Fickian release), 0.89 < *n* < 1 indicates a relaxation-controlled release, and in the case of *n* > 1, the release is controlled by swelling and chain relaxation [53,54,55,56].

**Figure 9:**
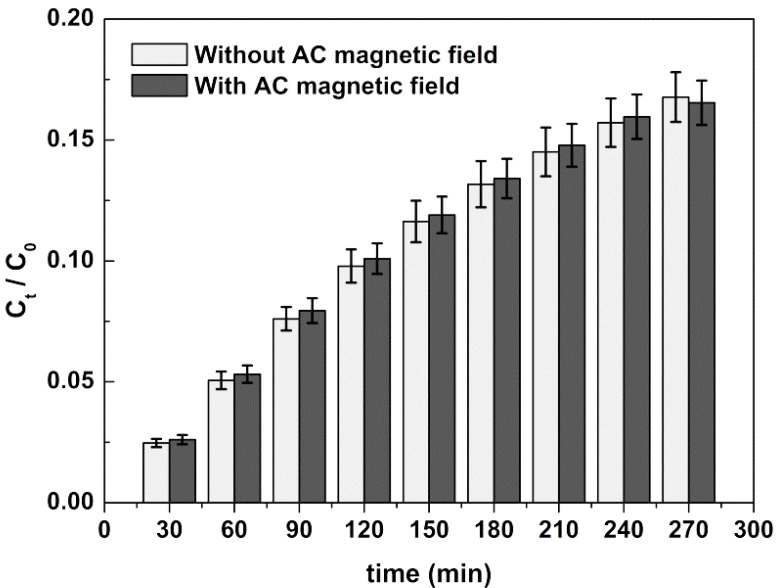
Drug release profile of curcumin from DPPC SMLs with and without the application of an alternating magnetic field. Experimental data is presented as average value ± standard deviation of three independent assays.

An enhanced release of curcumin from SMLs of DPPC was observed upon the application of an AC magnetic field (AMF) by 1.25 times, *K* = 2.98 × 10^−3^ min^−1^ and *K* = 3.73 × 10^−3^ min^−1^ in the absence and presence of AMF, respectively (Figure 9, Table 8). The local heating promoted by the magnetic nanoparticles makes the liposomes bilayer more fluid, with increased drug release. The estimated *n* values point to non-Fickian release (combination of diffusion and erosion) [57]. 

The curcumin release profile from PEGylated SMLs does not show a higher rate consistent with AMF application, indicating that the PEG shield may hamper the thermosensitivity of DPPC. Nevertheless, curcumin release is ca. ten times faster in PEGylated systems (with or without AMF). The low maximum magnetization of the calcium-substituted magnesium ferrite nanoparticles is probably the main reason for a small effect of the AMF application on drug release.

## 3. Materials and Methods

Ultrapure water Milli-Q grade (MilliporeSigma, St. Louis, MO, USA) and spectroscopic grade solvents were used in all preparation steps.

### 3.1. Preparation of Ca_x_Mg_1−__x_Fe_2_O_4_ Ferrite Nanoparticles

Calcium-substituted magnesium ferrite nanoparticles (Ca*_x_*Mg_1−*x*_Fe_2_O_4_) were prepared by coprecipitation method. First, an aqueous solution containing magnesium sulfate, hydrated calcium acetate at the corresponding molarity (for *x* = 0.25, 0.50 or 0.75), and iron (III) chloride hexahydrate was prepared, in a 1:2 molar ratio. The resulting mixture was added, drop by drop, to a 90 °C heated solution of sodium hydroxide at 5 M, under constant and vigorous magnetic stirring. The precipitated nanoparticles were washed by several cycles of centrifugation (5000 rpm for 5 min) and redispersed in water. Finally, the nanoparticles were calcined at 600 °C for 30 min. 

### 3.2. Preparation of Magnetoliposomes and GUVs

For aqueous magnetoliposome (AML) preparation, where magnetic nanoparticles are encapsulated in liposomes, an ethanolic solution of egg yolk–phosphatidylcholine (Egg–PC, from Sigma-Aldrich, St. Louis, MO, USA), was injected, under vortex, onto an aqueous solution containing 0.1 mM of nanoparticles, to a final lipid concentration of 1 mM (ethanolic injection method [58,59]). Then, the ferrofluid was washed with water by magnetic decantation to eliminate the nonencapsulated nanoparticles. Curcumin was incorporated into AMLs by the coinjection method, as previously described [22].

The preparation method of solid magnetoliposomes (SMLs), consisting in a lipid bilayer surrounding a cluster of magnetic nanoparticles, was previously developed by us and the formation of the liposomal structure has been confirmed in previous works [22,24], using the lipid DPPC (1,2-dipalmitoyl-*sn*-glycero-3-phosphocholine, from Sigma-Aldrich, St. Louis, MO, USA). First, 10 μL of a solution of magnetic nanoparticles (0.02 mg/mL) were ultrasonicated for one minute at 189 W, and 3 mL of chloroform was added to the solution, resulting in the formation of nanoparticle clusters. To form the first lipid layer, the solution containing the clusters was heated up to 55 °C (above the melting temperature of DPPC, 41 °C [46]) and 150 µL of a lipid methanolic solution (20 mM) was injected under vortexing. Magnetic decantation and several washing steps with ultrapure water were performed for purification, removing lipid aggregates and liposomes without a magnetic core. For the second lipid layer formation, an aqueous solution of the purified systems (3 mL) was heated up to 55 °C and 150 µL of a lipid methanolic solution (20 mM) was injected under vortexing. The resulting solid magnetoliposomes were then washed and purified with ultrapure water by centrifugation. Curcumin was incorporated by injection of an ethanolic solution, under vortexing, right before the formation of the second lipid layer.

Giant Unilamellar Vesicles (GUVs) were used as models of cell membranes and were prepared using a procedure previously described [44,45], using a 1 mM solution of L-α-phosphatidylcholine (Soybean lecithin, from Sigma-Aldrich, St. Louis, MO, USA).

### 3.3. Characterization of Nanoparticles and Magnetoliposomes

Magnetization measurements were carried out in a MPMS3 SQUID magnetometer MPMS5XL (Quantum Design Inc., San Diego, CA, USA). The hysteresis cycles (magnetization versus magnetic field) of the samples were measured in the convenient field range for each sample. The measurement method was by DC extraction or VSM oscillation at a frequency of 14 Hz. A specific magnetic field correction for the trapped flux in the superconducting coil was made achieving an accuracy of residual less than 2 Oe.

X-Ray diffraction (XRD) analyses were performed in a conventional Philips PW 1710 (Royal Philips, Amsterdam, The Netherlands) diffractometer, operating with CuK_α_ radiation, in a Bragg–Brentano configuration.

HR-TEM images were obtained at C.A.C.T.I (Centro de Apoio Científico e Tecnolóxico á Investigación), Vigo (Spain) using a Transmission Electron Microscope JEOL JEM 2010F (JEOL Ltd., Tokyo, Japan) operating at 200 kV. TEM images were processed using ImageJ software, version 1.52 p (National Institutes of Health (NIH), Bethesda, MD, USA).

Hydrodynamic diameters, polydispersity, and zeta potential values were determined in a Dynamic Light Scattering (DLS) equipment NANO ZS Malvern Zetasizer (Malvern Panalytical Ltd., Malvern, UK) at 25 °C, performing five independent measurements for each sample.

### 3.4. Spectroscopic Measurements

Absorption spectra were obtained in a Shimadzu UV-3101PC UV–Vis-NIR (Shimadzu Corporation, Kyoto, Japan) spectrophotometer. Fluorescence spectra were recorded in a Fluorolog 3 spectrofluorimeter (HORIBA Jobin Yvon IBH Ltd., Glasgow, UK), all spectra being corrected for the instrumental response. The steady-state fluorescence anisotropy, *r*, was measured in the latter equipment, using Glan–Thompson polarizers.

FRET assays were carried out as previously reported [22,24,41], allowing confirming the formation of the double lipid layer in SMLs and the interactions with model membranes. The detailed procedure is described in the Supplementary Material. The fluorescence quantum yield of the dye NBD (donor) in magnetoliposomes (containing the different magnetic nanoparticles) was determined by the standard method [60,61], using the labeled lipid NBD-C_12_-HPC in lipid membranes as reference, with Φ_r_ = 0.32 at 25 °C [62].

### 3.5. Drug Release Studies

In total, 0.5 mL of curcumin-loaded magnetoliposomes solution was placed in Amicon^®^ Ultra-0.5 mL centrifugal filters with 0.1 µm pore size (under mild shaking conditions) containing a GUVs solution in the bottom container, with GUVs being used as acceptor membrane models for curcumin. At 30 min intervals (for 4.5 h), 200 µL were collected from the acceptor compartment for assessing curcumin concentration, and an equal volume of fresh GUVs were added. The fluorescence of the collected solution was measured and the cumulative release of curcumin was calculated using calibration curves previously obtained. Three independent release assays were performed.

Since magnetoliposomes were envisioned as magnetic responsive nanosystems, the release profile of curcumin was also assessed under the actuation of an AMF. The AMF was generated in a custom-designed solenoid device (800 turns per meter, length: 31 cm and internal diameter: 4.8 cm) by applying an alternating electric current. A magnetic field of 2.98 mT at 1000 kHz was used. Release profile assays were performed in triplicate.

## 4. Conclusions

Magnetoliposomes, both aqueous and solid, containing calcium-substituted magnesium ferrite nanoparticles and sizes below 160 nm, were developed for the first time. Stealth (PEGylated) magnetoliposomes were also prepared. The solid nanosystems were tested as magnetic-responsive carriers for curcumin. Although the anticancer efficacy of curcumin is yet to be completely demonstrated, it has been reported that curcumin-based therapies can be excellent adjuvants of classic anticancer therapy. The non-PEGylated nanosystems showed to be promising as magnetic-responsive drug nanocarriers, while the PEGylated ones did not respond efficiently to AMF application, but exhibited enhanced curcumin release (with and without AMF). The developed magnetoliposomes are promising as carriers for curcumin in future therapeutic applications, making it possible to choose systems with enhanced drug release (PEGylated systems) or systems with increased release by AMF (non-PEGylated systems). Future work will involve the development of calcium/magnesium mixed ferrites with a higher saturation magnetization for efficient drug release upon application of an alternating magnetic field. 

## Figures and Tables

**Table 1:** Selected Rietveld analysis parameters.

Sample	O_x,y,z_	Micro Absorption Correction (#)		Overall Temperature Factor, B_over_	Lattice Constant (nm)	Size (nm)	R_f_	χ^2^
Ca_0.50_Mg_0.50_Fe_2_O_4_	0.378	No		0 (^+^)	0.839045	6.3	5.92	1.39
Ca_0.50_Mg_0.50_Fe_2_O_4_	0.377	No		−3.67	0.839196	6.4	3.19	1.23
Ca_0.50_Mg_0.50_Fe_2_O_4_	0.370	P_0_ = 0.69	τ = 0.14	0 (^+^)	0.839164	6.4	2.64	1.23
Ca_0.25_Mg_0.75_Fe_2_O_4_	0.372	P_0_ = 0.71	τ = 0.11	0 (^+^)	0.838978	5.8	3.62	1.26
Ca_0.75_Mg_0.25_Fe_2_O_4_	0.384	P_0_ = 0.72	τ = 0.09	0 (^+^)	0.843550	7.0	3.71	1.28

(#) Equation (8) in [27] with C = 1. (^+^) fixed values.

**Table 2:** Saturation magnetization (M_s_), remnant magnetization (M_r_), ratio M_r_/M_s_, and coercive field (Hc) of calcium-substituted magnesium ferrite nanoparticles.

Nanoparticles	Ms (emu/g)	Mr (emu/g)	Mr/Ms	Hc (kOe)
Ca_0.75_Mg_0.25_Fe_2_O_4_	8.83	0.08	0.009	5.74
Ca_0.50_Mg_0.50_Fe_2_O_4_	15.63	0.07	0.004	5.33
Ca_0.25_Mg_0.75_Fe_2_O_4_	12.98	0.15	0.011	5.45

**Table 3:** Hydrodynamic size, polydispersity, and zeta potential of egg–phosphatidylcholine (Egg–PC) aqueous magnetoliposomes containing calcium-substituted magnesium ferrite nanoparticles. (SD: standard deviation; PDI: Polydispersity index).

Nanoparticles	Hydrodynamic Diameter ± SD (nm)	PDI	Zeta Potential ± SD (mV)
Ca_0.25_Mg_0.75_Fe_2_O_4_	147.8 ± 21	0.15 ± 0.018	−16.8 ± 0.5
Ca_0.50_Mg_0.50_Fe_2_O_4_	149.2 ± 22	0.20 ± 0.013	−18.0 ± 0.8
Ca_0.75_Mg_0.25_Fe_2_O_4_	131.4 ± 19	0.22 ± 0.015	−15.3 ± 1.5

**Table 4:** Steady-state fluorescence anisotropy (*r*) values for curcumin at 25 °C in aqueous magnetoliposomes containing Ca_0.25_Mg_0.75_Fe_2_O_4_. Values for curcumin in liposomes and glycerol are shown for comparison.

System	Lipid Formulation	*r*
Liposomes	Egg–PC	0.340 [22]
AMLs	Egg–PC	0.320
AMLs	95% Egg–PC/5% DSPE-PEG2000	0.335
Glycerol	----	0.365 [22]

**Table 5:** FRET efficiencies (Ф_FRET_), Förster radii (R_0_) and donor–acceptor distances (r_AD_) calculated for the formation of the lipid bilayer in solid magnetoliposomes.

Nanoparticles	Φ_FRET_	*R*_0_ (nm)	*r*_AD_ (nm)
Ca_0.25_Mg_0.75_Fe_2_O_4_	0.87	5.31	3.87
Ca_0.50_Mg_0.50_Fe_2_O_4_	0.96	5.94	3.42
Ca_0.75_Mg_0.25_Fe_2_O_4_	0.68	3.59	3.17

**Table 6:** Hydrodynamic size, polydispersity, and zeta potential of DPPC solid magnetoliposomes containing calcium-substituted magnesium ferrite nanoparticles (SD: standard deviation; PDI: polydispersity index).

Nanoparticles	Hydrodynamic Diameter ± SD (nm)	PDI	Zeta Potential ± SD (mV)
Ca_0.25_Mg_0.75_Fe_2_O_4_	127.3 ± 17	0.213 ± 0.025	−19.8 ± 1.2
Ca_0.50_Mg_0.50_Fe_2_O_4_	147.0 ± 15	0.206 ± 0.012	−21.4 ± 3
Ca_0.75_Mg_0.25_Fe_2_O_4_	155.4 ± 21	0.228 ± 0.021	−20.9 ± 1.9

**Table 7:** Steady-state fluorescence anisotropy (*r*) values for curcumin at 25 °C and 55 °C in solid magnetoliposomes containing Ca_0.25_Mg_0.75_Fe_2_O_4_. Values for curcumin in DPPC liposomes are also shown for comparison.

	Lipid Formulation	Temperature (°C)	*r*
Liposomes	DPPC	25	0.287 [22]
		55	0.119 [22]
SMLs	DPPC	25	0.156
		55	0.094
SMLs	95% DPPC/5% DSPE-PEG2000	25	0.209
		55	0.139

**Table 8:** Parameters of the Korsmeyer–Peppas model for the release of curcumin from SMLs in the presence and absence of an AMF.

Conditions	Lipid Formulation	*K* (min^−1^)	*n*	*R* ^2^
Without AMF	DPPC	2.98 × 10^−3^	0.685	0.9929
	DPPC:DSPE-PEG	2.43 × 10^−2^	0.659	0.9927
With AMF	DPPC	3.73 × 10^−3^	0.646	0.9900
	DPPC:DSPE-PEG	2.26 × 10^−2^	0.663	0.9923

## Supplementary Materials

The following are available online at https://www.mdpi.com/1422-0067/21/10/3641/s1, Figure S1: Fluorescence emission spectra (λ_exc_ = 470 nm) of solid magnetoliposomes based on calcium-substituted magnesium ferrite nanoparticles labeled with only NBD-C_12_-HPC (donor), with only Rhodamine B-DOPE (acceptor) and labeled with both NBD-C_12_-HPC and Rhodamine B-DOPE. Equations (S1)–(S4): Equations for calculation of FRET efficiency and donor-acceptor distances.
