# Supplementary material for: Stealth Magnetoliposomes Based on Calcium-Substituted Magnesium Ferrite Nanoparticles for Curcumin Transport and Release
Source: Int J Mol Sci. 2020 May 21;21(10):3641. doi: 10.3390/ijms21103641 (PMC7279386; doi:10.3390/ijms21103641)
Supplement: Supplementary file 1 [file ijms-21-03641-s001.pdf]

Stealth magnetoliposomes based on calcium-substituted magnesium ferrite nanoparticles for curcumin transport and release

Beatriz D. Cardoso[1], Ana Rita O. Rodrigues[1], Bernardo G. Almeida[1], Carlos O. Amorim [2], Vítor S. Amaral [2], Elisabete M. S. Castanheira[1] and Paulo J. G. Coutinho [1,*]

[1] Centre of Physics (CFUM), University of Minho, Campus de Gualtar, 4710-057 Braga, Portugal
[2] Physics Department and CICECO, University of Aveiro, Campus de Santiago, 3810-193 Aveiro, Portugal
* Correspondence: pcoutinho@fisica.uminho.pt

Supplementary Material

FRET measurements and equations

FRET efficiency, Φ_{RET}, defined as the proportion of donor molecules that have transferred their excess energy to acceptor molecules, can be obtained by taking the ratio of the donor integrated fluorescence intensities in the presence of acceptor (F_{DA}) and in the absence of acceptor (F_D) (equation S1),

$$\Phi_{\mathrm{RET}} = 1 - \frac{F_{\mathrm{DA}}}{F_{\mathrm{D}}} \tag{S1}$$

The distance between donor and acceptor molecules can be determined through the FRET efficiency (equation S2),

$$r_{\mathrm{AD}} = R_0 . \left[\frac{1-\Phi_{\mathrm{RET}}}{\Phi_{\mathrm{RET}}}\right]^{1/6} \tag{S2}$$

where R_0 is the Förster radius (critical distance), that can be obtained by the spectral overlap, $J(\lambda)$, between the donor emission and the acceptor absorption, according to equations (S3) and (S4) (with R_0 in Å, λ in nm, $\varepsilon_A(\lambda)$ in M^{-1} cm^{-1}),

$$R_0 = 0.2108 \left[k^2 \Phi_D^0 n^{-4} J(\lambda)\right]^{1/6} \tag{S3}$$

$$J(\lambda) = \int_0^\infty I_D(\lambda) \varepsilon_A(\lambda) \lambda^4 d\lambda \tag{S4}$$

where $k^2 = 2/3$ is the orientational factor assuming random orientation of the dyes, Φ_D^0 is the fluorescence quantum yield of the donor in the absence of energy transfer, n is the refraction index of the medium, $I_D(\lambda)$ is the fluorescence spectrum of the donor normalized so that $\int_0^\infty I_D(\lambda) d\lambda = 1$, and $\varepsilon_A(\lambda)$ is the molar absorption coefficient of the acceptor.

FRET assay for the formation of the lipid bilayer in SMLs

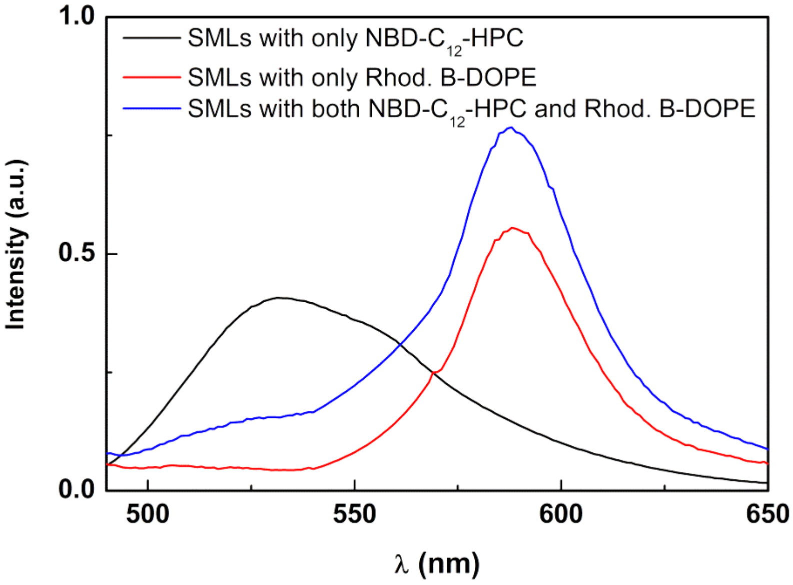

Figure S1. Fluorescence emission spectra (λ_{exc}=470 nm) of solid magnetoliposomes based on calcium-substituted magnesium ferrite nanoparticles labeled with only NBD-C_{12}-HPC (donor), with only Rhodamine B-DOPE (acceptor) and labeled with both NBD-C_{12}-HPC and Rhodamine B-DOPE.
